# The Practical Work of Ensuring the Effective Use of Serious Games in a Rehabilitation Clinic: Qualitative Study

**DOI:** 10.2196/15428

**Published:** 2020-02-28

**Authors:** João Almeida, Francisco Nunes

**Affiliations:** 1 Fraunhofer Portugal AICOS Porto Portugal

**Keywords:** serious games, exergames, physiotherapy rehabilitation, practical rehabilitation work, qualitative research

## Abstract

**Background:**

Many rehabilitation clinics adopted serious games to support their physiotherapy sessions. Serious games can monitor and provide feedback on exercises and are expected to improve therapy and help professionals deal with more patients. However, there is little understanding of the impacts of serious games on the actual work of physiotherapists.

**Objective:**

This study aimed to understand the impact of an electromyography-based serious game on the practical work of physiotherapists.

**Methods:**

This study used observation sessions in an outpatient rehabilitation clinic that recently started using a serious game based on electromyography sensors. In total, 44 observation sessions were performed, involving 3 physiotherapists and 22 patients. Observation sessions were documented by audio recordings or fieldnotes and were analyzed for themes using thematic analysis.

**Results:**

The findings of this study showed that physiotherapists played an important role in enabling the serious game to work. Physiotherapists briefed patients, calibrated the system, prescribed exercises, and supported patients while they played the serious game, all of which amounted to relevant labor.

**Conclusions:**

The results of this work challenge the idea that serious games reduce the work of physiotherapists and call for an overall analysis of the different impacts a serious game can have. Adopting a serious game that creates more work can be entirely acceptable, provided the clinical outcomes or other advantages enabled by the serious game are strong; however, those impacts will have to be assessed on a case-by-case basis. Moreover, this work motivates the technology development community to better investigate physiotherapists and their context, offering implications for technology design.

## Introduction

### Motivation and Overview

Demographic changes in the last few decades have been challenging physiotherapists and health care institutions in Western countries. As people age, they are more prone to falls, strokes, and cardiac diseases [1], all of which can trigger the need for physical rehabilitation and add pressure on rehabilitation clinics to deal with more patients. In the context of full-service clinics and multitasking professionals, serious games for physical rehabilitation were seen as a way to improve therapy and help physiotherapists deal with an increasing number of patients.

Serious games are game systems with nonentertainment purposes [2] that can be used to support or motivate activities, in this case, physical rehabilitation. Serious games are not new in the rehabilitation context and have been developed to (1) increase therapy dosage [3-5], (2) engage patients in activities that motivate them to persist in therapy [6-8], or (3) enable correct exercise performance at home [4,9,10]. Reading the literature on serious games for rehabilitation, we get the idea that physiotherapists would be lightly involved if at all in serious games [10,11] and that they would even be free to attend more patients [12,13]. In this vision, the therapist would still “attach the technology to the patient, and/or to operate the technology” [11], but serious games would continue the therapeutic intervention from there.

The vision that serious games would not require physiotherapists or even remove work from these therapists seemed to be too idealistic. We know from other health care settings that technology does not usually remove work but rather redistributes and reshapes existing activities [14]. Thus, we were curious to understand how the work of physiotherapists was impacted with the introduction of serious games.

This paper describes how physiotherapists set up and accompany the execution of a serious game based on electromyography sensors. Drawing on insights from 44 observation sessions conducted in an outpatient rehabilitation clinic, we argue that physiotherapists who used the serious game performed numerous activities that amounted to relevant work.

The contribution of this paper is two-fold. First, it presents an ethnographic description of the work of physiotherapists in setting up and supporting the execution of a serious game in a rehabilitation clinic, which shows labor and an active role from these professionals. Second, the paper offers design implications that follow from recognizing the work of physiotherapists in supporting serious games. We expect to inspire the technology development community to better account for the role and work of physiotherapists when designing serious games for rehabilitation. Moreover, we try to ensure physiotherapy professionals are aware that serious games can require an active role from them to achieve the promised benefits to engagement or intervention efficacy.

### Background

The literature on serious games for rehabilitation, which includes exergames, virtual reality, or interactive video games, has been growing in the past years [15]. We know as a community that serious games can help treat conditions such as Parkinson disease [10,16] and stroke [17,18] and help improve balance or exercise for different patients [19,20]. Most publications on serious games have focused on designing or initially assessing the impact of serious games [15]. As serious games aimed to support therapy, many studies focused on assessing medical outcomes and the quality of the exercise performed with the technological systems [16,17,20]. Qualitative studies tended to focus on the experiences of patients using serious games in controlled settings or at home [10,19,21].

The experiences of physiotherapists with serious games received little attention. The few studies that assessed the experience of therapists with serious games in clinics mention that they play a role in setting up [22,23], training [24], providing feedback or assistance during the games [24], and cleaning up or maintaining systems [22]. Although these activities are mentioned in some studies, there is little detail about what physiotherapists actually do and the impact it has on their overall work. This is especially concerning because according to Markus et al [22], who timed different activities of physiotherapists in setting up and playing serious games in a burn care unit, playing the game accounted for solely 22% of the time of the therapists, whereas setting up, training, cleaning, and maintaining the system occupied the remaining time.

The role of therapists in serious games for home rehabilitation is also rarely discussed. Some papers mention that physiotherapists are involved in setting up the game [25] or instructing patients to perform the game [4], but most papers we find seem to expect a reduced role from physiotherapists [10,16,21]. Although the patient can be instructed and monitored by a serious game, the initial diagnosis and follow-up assessments are most likely performed by a physiotherapist. Thus, we believe that the work of physiotherapists in this setting is somehow unacknowledged or hidden.

Although prior work evaluating serious games paid little attention to the work and role of physiotherapists, studies discussing the perspectives of physiotherapists on these interventions painted a different picture. Drawing on focus groups or workshops with physiotherapists, different studies argue that therapists would likely be required to set up the system for patients, which was a concern as therapists are often overloaded with different activities [26,27]. The same studies concluded that therapists would need to reserve time to learn to use a serious game and test on themselves, to know how to orient patients in clinical practice. Moreover, studies point to the expectation of having therapists involved in personalizing exercise for the patients [3,8,27]. According to these works, therapists would be the ones choosing exercises, difficulty, and tools that better fit the characteristics and interests of the patients.

On the whole, there is a reduced understanding of the role and work of physiotherapists in enabling serious games. Although some studies mentioned that therapists were involved in activities, what therapists did is mostly hidden. This paper will help address this issue by discussing the practices of physiotherapists in enabling serious games.

## Methods

### Overview

To understand how physiotherapists set up and use a serious game in their clinical practice, we observed physiotherapy sessions in an outpatient rehabilitation clinic. The observation was conducted by the first author, who ranged from being a spectator not intervening during physiotherapy sessions with patients to actively inquiring patients and therapists once the rehabilitation session was finished. The observation took place in the clinic’s gymnasium, where 2 to 3 physiotherapists care for a set of patients at the same time. The gymnasium was well equipped for supporting physiotherapy sessions, including examination beds, Pilates balls, treadmills, weights, and computers, in addition to the serious game we were studying. The outpatient rehabilitation clinic was part of a large public rehabilitation center located in the north of Portugal.

The initial goal of the observation was to understand how patients, carers, and therapists used the serious game in clinical practice, but as the study advanced, we started focusing on the practical work that was required to make the system work. As part of the fieldwork, we also conducted interviews with patients to understand their experience with the serious game, but that is out of the scope of this paper.

In total, 44 observation sessions were performed with 22 patients and 3 physiotherapists. The physiotherapists, 1 male and 2 females, had a Master’s degree in Physiotherapy and 11 to 15 years of experience in rehabilitation (see Table 1). None of the physiotherapists had experience with electromyography- or sensor-based interventions before experiencing eleRehab; however, they had used the Wii Fit with some patients in the past. With regard to technology use, all physiotherapists had smartphones, and there were computers in the gym to support some interventions, so we are led to believe that the physiotherapists were receptive to using digital technologies in their personal and professional lives. Before using the system in clinical practice, the 3 physiotherapists received multiple sessions of professional training from a physiotherapist experienced in using eleRehab who worked for the company that developed a part of eleRehab. When we observed the physiotherapists, they were already able to use the system in clinical practice.

The recruitment of the patients was performed by their physiotherapist, taking into consideration the characteristics of the patient, their ongoing intervention plan, and the fit of the system to the rehabilitation plan. There were 12 male and 10 female participants. No participant had university training, some had high school diplomas, and others only attended primary school education. Their ages ranged from 21 to 58 years, and they were doing physical rehabilitation to recover functionality and return to their work and everyday lives (see Table 2). The patients neither had experience with electromyography games nor usually played games regularly in their free time. Most patients had smartphones, but participants were not heavy technology users, restricting their use to a small number of apps.

We conducted a total of 44 observation sessions. The first 20 observation sessions were audio recorded to enable detailed analysis. After 20 sessions, we achieved meaning saturation [28] but continued observation sessions, making fieldnotes to comply with project objectives. The sessions with eleRehab lasted between 60 and 90 min (average 78 min), and we recorded a total of 26 hours of audio recordings. Audio recordings were transcribed verbatim, enriched with fieldnotes, and coded for themes using thematic analysis [29]. We tried to remain as open as possible to the themes that were salient in the data and, thus, coded the different observation sessions iteratively. Moreover, we leveraged constant comparison [30] to advance the analysis, making use of the differences between observation instances, patients, and physiotherapists. The Scrivener writing software (Literature & Latte) supported the coding process.

Regarding ethics, we obtained written informed consent from all physiotherapists and patient participants. In each case, we started by presenting the researchers involved, the project and its goals, and the reasons for the observation. We cleared any doubts the participants could have, and only then did the participants sign the informed consent form.

**Table 1:** Characteristics of physiotherapists.

Physiotherapist	Age (years)	Gender	Work experience (years)	Experience with electromyography
Physiotherapist 1	34	Female	11	None
Physiotherapist 2	33	Male	11	None
Physiotherapist 3	36	Female	15	None

**Table 2:** Characteristics of patients involved.

Patient	Age (years)	Gender	Rehabilitation trigger	First session	Second session
Patient 1	47	Male	Myocardial infarction	Physiotherapist 1	Physiotherapist 2
Patient 2	20	Female	Spina bifida	Physiotherapist 1 and Physiotherapist 2	Physiotherapist 3
Patient 3	28	Male	Stroke	Physiotherapist 2	Physiotherapist 1
Patient 4	58	Male	Stroke	Physiotherapist 2	Physiotherapist 2
Patient 5	44	Female	Poliomyelitis and sciatica	Physiotherapist 1	Physiotherapist 1
Patient 6	35	Female	Stroke	Physiotherapist 2	Physiotherapist 3
Patient 7	56	Female	Head trauma	Physiotherapist 1 and Physiotherapist 2	Physiotherapist 3
Patient 8	42	Male	Stroke	Physiotherapist 1 and Physiotherapist 2	Physiotherapist 3
Patient 9	32	Female	—^a^	Physiotherapist 2	Physiotherapist 3
Patient 10	55	Male	Shoulder prosthetics	Physiotherapist 2	Physiotherapist 3
Patient 11	49	Female	Cervical prosthesis	Physiotherapist 1	Physiotherapist 2
Patient 12	37	Male	Dilated cardiomyopathy	Physiotherapist 1	Physiotherapist 2
Patient 13	49	Female	Breast cancer	Physiotherapist 1	Physiotherapist 2 and Physiotherapist 3
Patient 14	44	Male	Head trauma	Physiotherapist 1	Physiotherapist 2
Patient 15	40	Female	Breast cancer	Physiotherapist 1	Physiotherapist 2
Patient 16	47	Female	Spinal cord injury	Physiotherapist 2	Physiotherapist 2 and Physiotherapist 3
Patient 17	42	Male	Head trauma	Physiotherapist 2	Physiotherapist 2
Patient 18	44	Male	Cerebral angioma	Physiotherapist 2	Physiotherapist 2
Patient 19	51	Male	Stroke	Physiotherapist 3	Physiotherapist 3
Patient 20	47	Male	Cerebral angioma	Physiotherapist 3	Physiotherapist 3
Patient 21	29	Male	Head trauma	Physiotherapist 3	Physiotherapist 2
Patient 22	36	Female	Head trauma	Physiotherapist 3	Physiotherapist 2

^a^Missing data.

### The eleRehab System

The serious game we studied in the clinic, here named eleRehab, was targeted at the rehabilitation of muscles from the shoulder. Patients wore two sensors in the back and performed exercises in front of a smartphone, where a game was displayed. The games had elevating platforms, labyrinths, and opening gates, which *forced* patients to perform contractions and relaxations of their muscles for a certain period. In terms of environment requirements, the game was expected to be played in a well-lit room because of the small form factor of the smartphone screen, but there were no requirements regarding ambient noise, as the game featured no sound effects or music.

**Figure 1:**
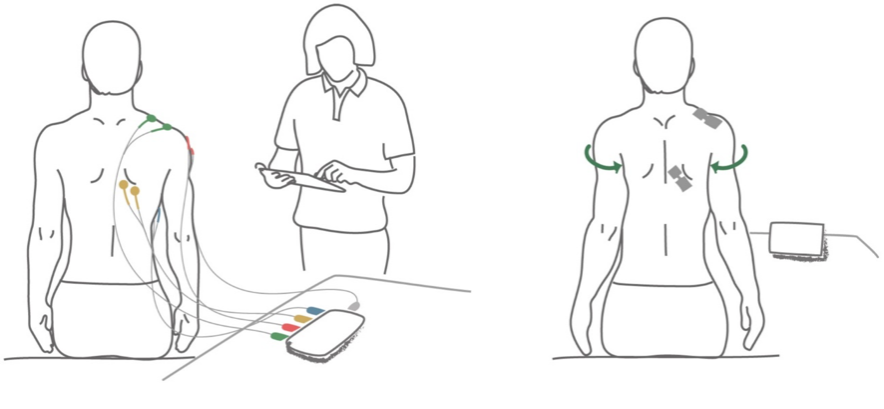
The eleRehab system. Left figure shows a physiotherapist calibrating the system for the patient using the calibration octopus and a tablet. Right figure shows the patient wearing sensors on the back and playing the game on the smartphone.

Physiotherapists had separate sensors for calibrating the system to the patient, the calibration octopus, and a tablet device for prescribing the number of series and exercises for each patient. The calibration octopus is named this way because it has 4 cables that divide into 8 electrical leads (see left part of Figure 1). Communication between sensors and tablet/smartphone is performed using Bluetooth, and the electronic prescriptions of exercises were stored in the cloud. In a typical usage of the eleRehab, physiotherapists calibrated the system for a specific patient using the calibration octopus and prescribed exercises with their tablet. Only after this, would they attach sensors to the back of the patient and have them play the serious game (see right part of Figure 1).

eleRehab explores electromyography or the measurement of electric current from the muscles. Each time we move our muscles, we send an electric charge from the brain to the muscle, and the current is stronger when we apply more strength to an exercise. The difference in current measured at a particular muscle enables eleRehab to know when the person is flexing or relaxing the muscle, and in this way, the system can monitor and provide feedback on the execution of exercises to the patient.

Most components of the system were developed by a medical device company that creates sensor-based tools for physiotherapy clinics. The game itself was designed by a team at Fraunhofer Portugal AICOS, of which the authors are part of. The development of the system followed a user-centered design approach with multiple phases of design, usability testing, and pilots. The results presented in this paper refer solely to the evaluation of the overall system in the clinic.

## Results

The fieldwork presented here describes the practical work required to set up and play eleRehab in a rehabilitation clinic. We describe four main themes or activities, namely, briefing the patient, calibrating the system, prescribing exercises, and playing the serious game.

### Briefing the Patient

The physiotherapy sessions with the serious game started with the therapist explaining the treatment procedure to the patient. Therapists explained to patients that they would perform exercises using a serious game and that the session would have two parts. First, therapists would connect and calibrate sensors to personalize the game for the patient. Second, the patient would play the game while performing specific exercises. Therapists explained that the system could sense when their muscles contracted and relaxed and would use this information to control the game. However, it needed to be personalized to each person’s body and thus required calibration. The therapists also mentioned that the game would improve the mobility, strength, or coordination, depending on the issue they were treating and the patient’s case. As the system targeted shoulder rehabilitation, therapists politely asked the patient to undress the upper part of their body, as they would need to connect the calibration octopus sensors shortly after. The goal of briefing the patient was two-fold. The therapists wanted to explain the procedure to the patients so that they would be informed and feel in control of what was happening at the clinic. At the same time, the therapists felt that they had to explain the system to the patients to obtain an appropriate performance, as patients would better engage with the game if they understood how it worked and how to perform at their best.

### Calibrating the System

Calibrating sensors is a complex activity that is composed of several steps. The physiotherapist starts by creating a profile for the patient on their tablet. Therapists enter the name, email, and weight of the patient, and they signal the shoulder to be treated next. After creating an account, the profile is listed in the tablet app, and therapists can choose it when starting a rehabilitation session. In any case, physiotherapists usually went over the information of the patient’s profile to confirm it was updated.

The second step of calibration is to attach the calibration octopus sensors to the patient (left part of Figure 1). To do so, physiotherapists locate each muscle, attach 2 disposable electrodes to it, and connect 2 leads from the calibration octopus to the electrodes of the patient. Connecting the leads to the patient requires palpation and sometimes asking the patient to perform movements that *reveal* the muscle. This process can take some time when muscles are under adipose tissue or when they have irregular electric responses because of the lesion of the patient. eleRehab requires leads to be placed in the lower trapezius, upper trapezius, anterior deltoid, and anterior serratus. After attaching the leads to each muscle, the last one called *earth* lead is connected to the clavicle of the patient. The placement of the leads is performed with the aid of the tablet because they are numbered from 1 to 4, and each number is related to a specific muscle. Through the app, the physiotherapist knows to which muscle each number belongs. Moreover, the placement of the leads in each muscle needs to be within a fixed distance. When playing the game, patients will wear a sensor that has a fixed length, and if the leads of the calibration octopus are not distanced similarly, problems may arise during game execution. For this reason, physiotherapists place the leads of the calibration octopus in the muscle, at a distance that is the same as the distance they will have in the patients’ sensors (right image in Figure 1).

After connecting the calibration octopus, the physiotherapist is ready to measure the electric response of the muscles. Physiotherapists first measure the electric response of the muscles while performing specific exercises and then add resistance to capture the maximum electric response of the muscles. The patients performed three exercises: frontal arm extension, lateral arm extension, and diagonal arm extension. The physiotherapist explains and exemplifies each exercise and instructs the tablet app when to start collecting data. The app makes a sound to notify both the physiotherapist and the patient to start the exercise movement and, after that, collects data about the muscle’s electric response. The measurement of electric response is repeated when therapists believe the exercise was not correctly executed. During the first time therapists used eleRehab, they asked patients to repeat exercises multiple times to compare the electrical response of different trials. However, as they gained confidence that repeated measures yield similar values, therapists stopped asking patients to repeat exercises.

The muscle acquisition with resistance follows. This time, the physiotherapist asks patients to repeat the 3-arm extensions mentioned above, but this time, they apply force contrary to the movement of the patient. The goal of this collection is to find the maximum contraction values for each muscle, so therapists can prescribe exercises that are appropriate to the patient’s muscles.

Having performed the above-mentioned steps, the system is calibrated for that specific patient. The calibration process might be required some days later, as the maximum electric response of the muscles may change, aligned with one’s rehabilitation.

### Prescribing Exercises

**Figure 2:**
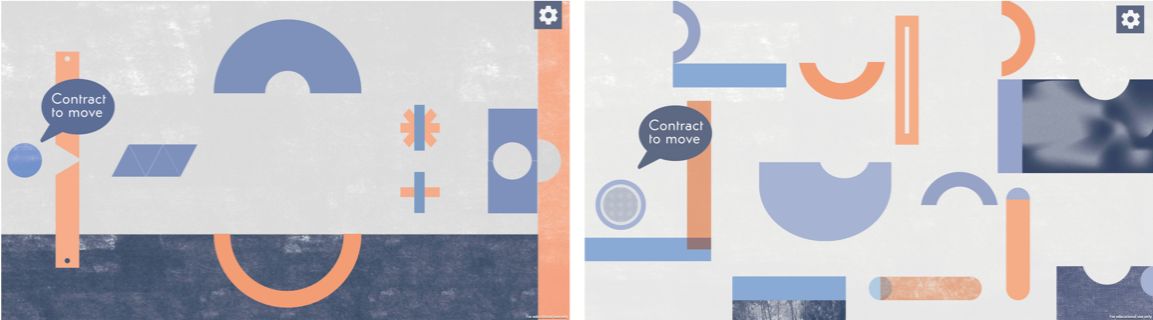
Screenshots from the serious game used by the patients. Left screen displays the game of the platforms, which opens gates as patients contract or relax muscles. Right screen displays a labyrinth where a ball is sent over the scenario as contractions and relaxations are performed at the right time.

Once the system is calibrated, physiotherapists can prescribe exercises for a patient. Physiotherapists first choose an exercise from a list and then ask patients to perform the exercise to personalize its characteristics. Although patients perform an exercise, therapists observe the contraction and relaxation values of the involved muscles and define upper and lower thresholds for exercises. For example, in an exercise where the patient pulls the shoulders back, as in the left image of Figure 2, patients will contract the lower trapezius and relax the upper trapezius and will have upper and lower thresholds to know when the muscle is contracted or relaxed. During the serious game execution, the person will be able to advance the game when their lower trapezius is above a particular threshold value and when their upper trapezius is below a particular threshold value. Thus, it is crucial that the values are appropriate for the patient. Moreover, and as mentioned before, thresholds may need to be updated as patients advance in their rehabilitation process.

After choosing thresholds, physiotherapists assess if they are appropriate for the patient. To do so, they ask the patient to perform an exercise for 10 seconds. If they are able to keep the muscles contracted/relaxed over/under a certain threshold, thresholds are appropriate. If patients cannot keep the exercise, the physiotherapist may ask the patient to repeat the exercise or adjust the thresholds. The idea is that the exercises slightly challenge patients, but they cannot become overexerted with effort as that can be detrimental to the rehabilitation.

The tablet app of the physiotherapist plays an essential role in adjusting thresholds. Each muscle has a bar that is updated in real time in the tablet app to reflect the increase or decrease of the electric response of the muscle. Moreover, the bar is green when the execution is under/over the expected threshold and red when that is not the case. Although the tablet app was thought as an assistant to the physiotherapist, it is often shown to the patient to improve the execution of the exercise (see Figure 2). Physiotherapists give tips to improve the execution, and whenever a bar turns red, the physiotherapist explains why and what was the problem in the execution of the exercise to enhance the autocorrection by the patient. Moreover, physiotherapists encourage patients to perform exercises incorrectly, so they can see bars getting red and learn how to correct their exercises by themselves.

Once thresholds are properly defined for each exercise, physiotherapists can change the number of sets, the number of repetitions, the execution time of each repetition, and the rest time between sets. They can also select the sensors to be used by the patient to play the game. Then, physiotherapists associate the prescription of the patient to an email address. Patients play the serious game in a smartphone by logging in with an email address. In principle, patients’ prescriptions would be associated with their email address, but during all therapy sessions, the prescriptions were sent to the same email address, the one configured on the smartphone of the clinic, to avoid log-in issues and speed up the process.

After finishing the prescription, the physiotherapist marks with a pen the muscles of the patient where the sensor should be attached.

### Using a Serious Game

**Figure 3:**
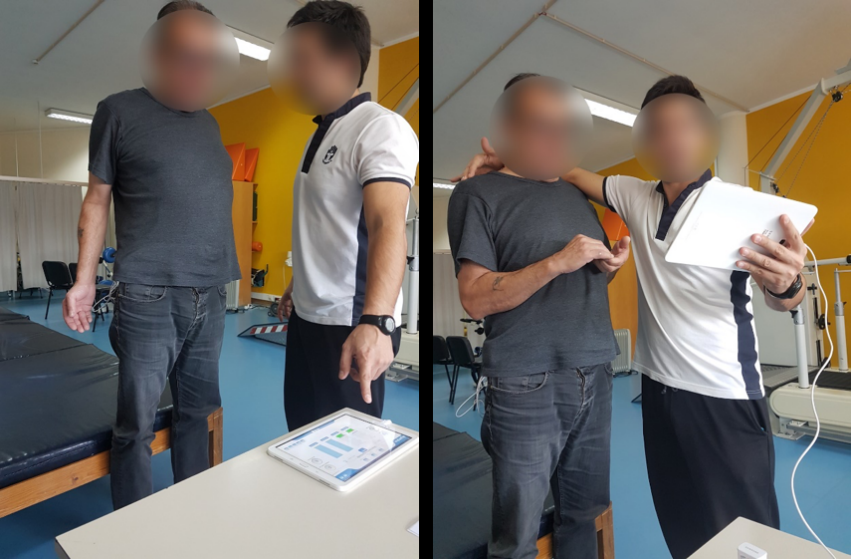
Physiotherapists often use their tablet application to explain to patients how to perform exercises correctly. Notice the calibration octopus in the pocket of the patient on both pictures, and the hand of the therapist correcting the exercise as the patient performs it, on the right image.

Performing a prescribed exercise also needs some preparation (see Figure 3). If the calibration octopus is still attached, physiotherapists need to remove the leads of the octopus and all the electrodes connected to the patient’s body. They also need to arrange a table, a mirror, and a support cushion for the patient to successfully play the serious game. Very often, physiotherapists will bring power plugs to connect the smartphone too, to avoid running out of battery while the patient plays the serious game.

Once these preparations have been pursued, physiotherapists remind the types of exercises patients will perform, what sensors they will attach to the body, how to turn on those sensors, and how patients will control the game in the end. Physiotherapists then open the smartphone app and hand the smartphone to the patient, so that they are proficient in running the system and, thus, are potentially able to use eleRehab at home.

The app begins by asking the patient to connect the smartphone to the sensors. Patients turn on the sensors, according to the physiotherapists’ instructions. Then the app shows the location of the muscle where to place the sensors. Patients usually try to place the sensors on their back by themselves, yet it can be difficult because of the location or their movement restrictions. The physiotherapist often corrected the placement of the sensors and asked if the patient had someone at home who could put the sensors on the marks made with the pen. The smartphone app then explains to the patient the exercise that they need to perform through a video and a textual description. Then, the game proceeds.

During the execution of the game, the therapist was often next to the patient observing the exercise execution. When patients played the game without difficulties, the physiotherapist did not intervene much, but if they faced difficulties in proceeding, the therapist would provide feedback on how to improve the exercise being performed. In some cases, the electrodes would detach, and the physiotherapist had to intervene again by placing the sensors in the muscle. The goal of the physiotherapist was to prepare patients to use the system at home autonomously; thus, they tried to refrain from intervening during the execution of the serious game.

When patients had more than one exercise prescribed, it was common to change the setup of the game. In these situations, the therapist was the one bringing other materials that were needed (eg, a Pilates ball, a step, or a cushion).

## Discussion

### Principal Findings

**Figure 4:**
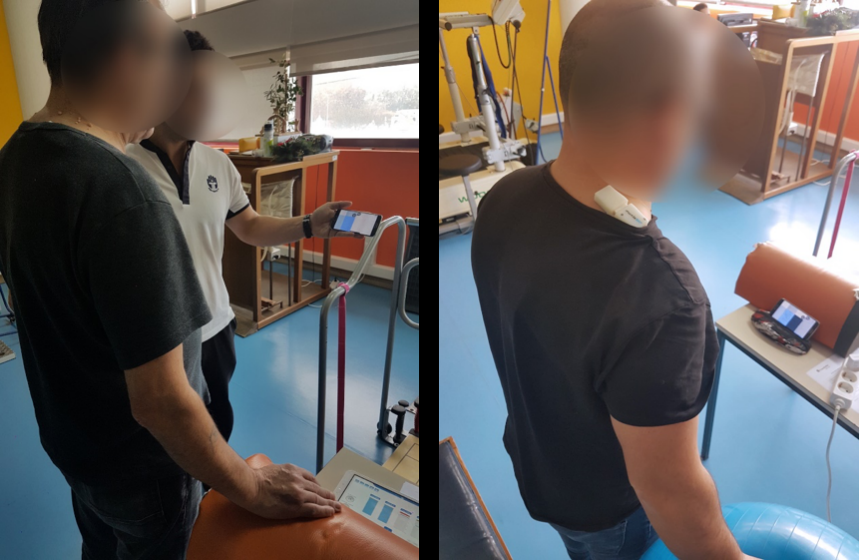
Patient playing the eleRehab serious game with a smartphone and two sensors worn on the back to monitor exercises. Notice the table, Pilates ball, and other materials supporting exercise execution and the active role of the physiotherapist in supporting the patient in playing the serious game.

The findings presented above show that physiotherapists played an important role in enabling the serious game eleRehab to work. Physiotherapists briefed patients, calibrated the system, prescribed exercises, and supported patients while they played the serious game (see Figure 4). These results challenge the idea that physiotherapists have a reduced role in enabling serious games or that these interventions would provide some free time for the professionals to attend more patients.

Setting up a system that draws on electromyography, such as eleRehab, can take more time than a serious game that relies on inertial sensors or cameras because of the time calibrating the system to detect the muscle response of the patient. However, we would still expect physiotherapists to be actively involved in monitoring exercises in serious games based on inertial sensors or cameras because these systems can have issues in assessing the quality of the performed exercises. In any case, we can conclude that serious games may give more work to the physiotherapist than what was initially expected, and therefore, understanding therapists’ work and practices is fundamental to create a system that suits the activities of these professionals.

The active role of physiotherapists in enabling serious games is not inherently negative. If a specific serious game helps increase therapy dosage, sustain motivation, and/or enable the correct performance of exercises, it can be completely worth using, even if the serious game requires physiotherapists to invest time in making it work. This means that the most important question to ask when assessing a serious game is whether it can yield improvements to the therapy activities, not if the serious game will free time for the physiotherapists to attend more patients.

The activities uncovered in this paper align with previous studies investigating the use of serious games in clinics, which argued that physiotherapists were involved in setting up, training, offering feedback, and maintenance [22-24]. To this body of work, we add that physiotherapists are involved in arranging elements in the space where support exercise activities are performed, such as getting tables, cushions, and balls. Moreover, we explained the steps that are involved in successfully achieving these categories of activities.

All patients played the game in the clinic, but they could have taken it home with them. In that situation, the physiotherapist would have taken care of the setup and prepared patients to perform the exercises in autonomy, as expected in previous work [4,25]. The participant role of physiotherapists in preparing home rehabilitation games challenges another accepted idea that patients set up and play rehabilitation games by themselves at home. Considering that therapists are needed to evaluate patients, prescribe therapies, and personalize exercises [3,8,27], it seems unlikely that a game would enable therapy out of the box. Thus, we may observe similar activities of setting up and training before patients start using a serious game at home.

### Limitations

This paper was based on observations of a small group of physiotherapists who recently started to use the eleRehab in their clinical practice. These professionals spent a long time setting up the system (typically three or four times the time spent playing), which would likely be reduced as these professionals gained experience in using the system or chose longer exercises/games in their practice. Moreover, the patients who were involved in the study were especially complex as they often presented irregular muscle responses, caused by the complex neurological consequences of the diseases they suffered from. It would be *easier* for professionals to calibrate the system for other patients. Nevertheless, because eleRehab depends on a calibration phase to measure the muscle response, it is likely that a moderate calibration period will always exist and require professionals to be actively involved in it.

The characteristics of the serious game we observed also had an impact on the results, as electromyography games require calibration of the sensors and prescription of the exercises is a requirement in electromyography-based games to enable the game to work properly for the patient. However, as we explained above, we would expect an active role and engagement of physiotherapists in serious games that did not include a systematic calibration, for example, to monitor the quality of the performed exercises.

### Implications

Recognizing the work of physiotherapists in making serious games work in practice has important implications for the design of these systems. We discuss the three most obvious implications: (1) accept that serious games may add work, (2) involve physiotherapists during the whole design process, (3) involve physiotherapists during the whole design process, and (4) focus on the practical activities and context of physiotherapists.

#### Accept That Serious Games May Add Work

Our fieldwork shows that the serious game added work to the physiotherapists. As therapists wanted to use eleRehab, they needed to engage in numerous activities to set up, calibrate, and run the system. eleRehab might have lengthy setup processes because it relies on electromyography to capture exercise execution, but other serious games are also likely to generate work for physiotherapists. By recognizing that serious games do not always reduce work, as is usually mentioned in the literature [22,26,27], the technology design community will be better able to provide a balanced perspective on the impact of serious games. Moreover, we will be better able to investigate the work burden of serious games, if we consider that there is a good chance that serious games will create work for those involved in setting up and using them.

#### Involve Physiotherapists During the Whole Design Process

The crucial role of physiotherapists in enabling eleRehab calls for a greater involvement of therapists in the design of serious games. Although the technology design community acknowledges the importance of learning from health care professionals when designing technologies for health care [31,32], the role of physiotherapists in the design of serious games seems to be restricted. For example, from the set of studies cited in this paper, most involved physiotherapists only when defining the concept or requirements of the games or selecting the exercises to include [6,7,11,21]. Other studies only include the physiotherapist in the last phases of the design of the system. For instance, Duarte et al [33] developed a serious game for rehabilitation, which also included a mobile interface for the physiotherapist to monitor and define game parameters without involving them from the beginning. Including physiotherapists at different points will ensure that serious games fit their activities and clinical processes in the best way possible, even if games end up adding some work activities. Moreover, therapists can be crucial in the acceptance and implementation of serious games in a clinical context as they set up and explain how to use systems to the patients.

#### Focus on the Practical Activities and Context of Physiotherapists

This paper offered some examples of strategies of physiotherapists to practically support the execution of the game. Using tables and cushions for supporting the smartphone, using the tablet visualizations for increasing knowledge of the patient about the game, and always employing the same email to avoid log-in issues were some examples of practical strategies. These insights remind us that there is much to learn about how physiotherapists use serious games in practice to inform the design of serious games. Theories on appropriation mention that the design of technologies does not end in the designer’s hands but rather in the way technology is appropriated in situ by its users [34]. By investigating physiotherapists’ practical activities and context, the technology design community should be inspired to support efficient ways of dealing with serious games in practice and, in this way, better design serious games for those contexts.

### Conclusions and Future Work

This paper described how physiotherapists made a serious game work in an outpatient rehabilitation clinic. It was clear that physiotherapists engaged in several activities to enable patients to use the technology successfully. Our results challenge the idea that serious games require a reduced role of physiotherapists, showing different activities people needed to do because they used the serious game eleRehab. Moreover, we present implications that can better shape serious games to fit physiotherapists’ work and context.

In the future, we will continue observing the usage of eleRehab. We will have a chance to interview patients and physiotherapists, and we plan to contrast their perspectives on the serious game, as it is implemented in that particular clinic. We will also investigate how patients and physiotherapists make the system work when they take it home with them.

Moreover, we see interest in investigating how other serious games are used in practice to understand which activities are commonly generated by serious games when they reach the clinic.
